# The Menn Phonetic Mini-Corpus: Articulatory Gestures as Precursors to the Emergence of Segments

**DOI:** 10.3389/fpsyg.2021.646090

**Published:** 2021-04-28

**Authors:** Lise Menn, Ann M. Peters, Yvan Rose

**Affiliations:** ^1^Institute for Cognitive Science and Linguistics Department, University of Colorado, Boulder, CO, United States; ^2^Linguistics Department, University of Hawaii, Manoa, HI, United States; ^3^Department of Linguistics, Memorial University of Newfoundland, St. John's, NL, Canada

**Keywords:** child phonology, phonological emergence, phonbank, childes, speech development, longitudinal case study, child language corpus, articulatory representation

## Introduction

The Menn Phonetic Mini-Corpus (MPMC) is a phonetically transcribed American English dataset now available from the PhonBank database at https://phonbank.talkbank.org/derived/. The MPMC consists of 5 h 22 min of detailed transcription in IPA (International Phonetic Alphabet) of the babble and early speech of a toddler at the one-word stage named Jacob, and his conversation partners, along with the corresponding downloadable audio files. The IPA transcription was made by a linguist with over 50 years of experience in transcribing early child speech; most other recorded corpora that are available to the research community are transcribed in conventional spelling or analyzed globally instead of being transcribed, and therefore cannot be searched for the occurrence of particular speech sounds and their context. Such phonetic searches provide entry points for acoustic analyses as well as for the descriptive analyses presented here.

The MPMC allows the study of the sounds and sound patterns of Jacob's babble and early speech over the first 3 months of his use of words, and comparison with his sound patterns during a 1-month period starting about 3 months later. Four analyses in section Illustrative Analyses below indicate some of the kinds of phonetic studies that can be done. Most importantly, an impressionistic gestural analysis of 60 variants of his word “down” indicates that Jacob has a pre-segmental articulatory representation of most of the word; the pre-segmental portion consists of poorly coordinated articulatory gestures that have not yet been cross-linked to form phonetic segments. The gestural analysis, although limited to what can be inferred from transcriptions, provides a conceptual handle on what it is that phonetic segments emerge *from*, supporting and clarifying early aspects of several theoretical approaches to the emergence of phonological units (e.g., Beckman and Edwards, 2000; Edwards et al., 2004; Hay et al., 2004; Inkelas and Rose, 2007; Vihman and Croft, 2007; Menn et al., 2013; McAllister Byun et al., 2016; Vihman, 2019).

The 7-month time span (1;00.15 to 1;07.17) also permits the study of the development of the child's behavioral routines (Bates et al., 1980; Peters and Boggs, 1986), the maturation of his conversational interaction patterns, and the semantic/pragmatic development of proto-words into adult-like words. Note that the corpus is pre-syntactic: it contains just a few gestalt utterances (Peters and Menn, 1993) modeled on adult phrases and a few sporadic two-word combinations.

The MPMC will remain available for study in its present form in the “derived” section of PhonBank. The transcription and coding of the rest of the recordings will gradually be added to the main Menn Corpus on PhonBank, of which the MPMC will remain a proper subset (about 5% of the total Menn Corpus material). PhonBank began in 2006 as a supplement to the long-standing CHILDES database (*CHI*ld *L*anguage *D*ata *E*xchange *S*ystem; https://childes.talkbank.org) for the areas of phonetics and phonology, through the Phon software program (https://www.phon.ca) for the building and analysis of phonetically transcribed data corpora such as the MPMC. The PhonBank database (https://phonbank.talkbank.org) was instituted in 2011 as a database separate from CHILDES within the larger TalkBank system for language research (https://talkbank.org). Like all corpora published within PhonBank, the MPMC corpus is available for analysis in both Phon and CHAT formats, the latter for use within the CLAN program which powers CHILDES and most of the remainder of the TalkBank system. Phon enables research based on both phonetic transcriptions and acoustic data measurements, assessing for example the overall shape of word forms as well as the behavior of specific speech sounds and sound combinations across different contexts within the word. Researchers may add their own coding to the existing annotations of corpora in CHILDES, and may run additional phonological analyses using Phon (Hedlund and O'Brien, 2004; Rose et al., 2006; Rose and MacWhinney, 2014; Hedlund and Rose, 2020).

## Methods

Jacob was studied as a typically developing first child of academic parents living in Cambridge MA. The recording investigator (author LM) served as the child's regular caregiver, audiotaping at least an hour per day for 3 days a week over 8.5 months from 1;00.08 to 1;08.22. The eight sessions selected for the MPMC are divided into two parts, “Early” and “Later,” with ~170 tokens of word attempts produced by the child in each part. The Early part contains data from five sessions spaced over 3 months from 1;00.15 to 1;03.22, totaling just over 4 h. The Later part contains data from three sessions spaced over 1 month from 1;06.18 to 1;07.17, totaling 1 h, 22 min. The total transcription time in the Later portion is shorter because the Later transcriptions focus on parts of the sessions in which the child was producing more speech and babble; the Early part was less selective. The Early part contains about 2,750 adult utterances and the Later part contains 550. The Early part also contains about 500 babble utterances (speech-like utterances without identifiable target); the Later part contains about 160 babble utterances.

The sessions in the MPMC were originally recorded in 1974-75 using a high quality reel-to-reel Tandberg tape deck and Sennheiser microphone. Most of the recordings were made under naturalistic conditions in the toddler's home; background noise therefore limits the sound quality. Other adults, including his mother, were occasionally present and interacted as friends familiar with the investigator and the child. The MPMC also contains part of one session in which a trained psychology doctoral student presented Jacob with means-ends and object permanence test tasks from the Užgiris and Hunt (1975) developmental scales (Menn and Haselkorn, 1976). Field notes were made on the spot and each session was originally transcribed within 48 h of recording. All living participants have given consent to have these materials shared through CHILDES.

The 1974-75 study (Menn, 1976) was very limited by current standards: reflecting the theoretical biases and technical limitations of the era, only the child's words and proto-words (defined as meaningful recurring forms created by the child: Bates, 1976; Menn, 1976) were transcribed phonetically. Babbled utterances were indicated, but rarely transcribed. Speech directed to the child was transcribed orthographically; adult-adult speech was only indicated. A handwritten IPA list of the child's attempts at words in each of the ninety-odd recorded sessions was provided in Menn (1976), but none of the transcribed material was computerized or machine searchable.

In 2009 the tape recordings were digitized and uploaded to CHILDES; in 2019, investigator LM began re-transcribing the digitized recordings into the machine-searchable CHAT format. The re-transcriptions in the MPMC include all sufficiently audible adult speech regardless of the addressee, and all the child's transcribable babble and word-based sound play as well as word and word-like productions.

To increase transcription accuracy and separation of overlapping speech, we used Praat^1^ and CLAN^2^ for item-by-item playback, reduced playback speed, and “eyeballing” spectrograms and waveforms to help with time-indexing and segmenting the speech signal. These digital tools revealed many babbled sounds and word-attempts that had been missed with the analog devices of the 1970's.

After the initial IPA transcription in CHAT format, author YR converted the files to Phon and compiled the quantitative and qualitative data analyzed in Section illustrative analyses. Adult words and phrases with high degrees of conversational reduction were transcribed in IPA by investigator LM; IPA versions of the rest of the adult speech were obtained automatically from an IPA dictionary of pronounced words (citation forms) built into Phon.

The child's utterance types have been coded in CHAT format, using existing CHAT categories as much as possible. By far the commonest types were babble BB (defined as articulated utterances without identifiable target), filled pause FP (closed-mouth conversational turns), and word-targeted WT. Word-targeted utterances were subdivided where possible into proto-words PWD (meaningful recurring idiosyncratic forms) and real words RWD. Other types noted were cooing COO (purely vocalic utterances; Stark, 1980), word play WP, gestalt GST (Peters, 1983), and onomatopoeic ONO. Word-targeted utterances were cross-coded as being imitated IMIT, retrieved from long-term memory LTM, self-repetition SREP, and unprompted self-correction SCOR (Researchers wishing to use these codes should recode a sample to check consistency).

We remind readers that phonetic transcriptions are discrete representations of an essentially continuous multi-dimensional auditory-acoustic space; intra-transcriber reliability for sub-phonemic details is necessarily modest, about 50% overall. Most discrepancies between transcriptions of a given utterance were found where the child's pronunciation was the least controlled (e.g., phones or phone combinations that were still emerging; see below). Re-transcribing a sample is recommended if researchers wish to put weight on fine details; time markings in the transcription delimit every utterance, making it easy to check each one against the on-line audio recordings.

## Illustrative Analyses

Here we report four illustrative Phon-aided analyses of the MPMC, examining the child's phonological progress (or lack of it) from 1;00.15 to 1;07.17.

### Change in the Relative Proportions of Babble, Proto-Words, and Adult-Like Words in the MPMC

Comparing the Early (1;00.15 to 1;03.22) to the Later (1;06.18 to 1;07.17) sessions, the mean proportion of babble decreases from 0.81 to 0.50 of the ~1,000 transcribed child utterances. Proto-word tokens decrease from 0.15 to 0.09, while real word tokens increase from 0.05 to 0.41. All three of these changes may be taken as measures of Jacob's gradual transition from babble and idiosyncratic proto-words toward communication based on adult word targets.

### Distribution of Consonants in the MPMC

The distributions of consonants in the Early and Later portions of the MPMC were computed using Phon. From the beginning of the Early period, Jacob produced [m] appropriately, but only as a carrier for intonational signaling in “hm” or “mm” utterances. Whether this utterance type should be counted as production of a phonetic segment [m] is unsettled, because it does not form a canonical consonant+vowel syllable (Oller et al., 1999).

Jacob used [d], a common first consonant (Stoel-Gammon, 1985; Menn and Vihman, 2011), frequently from 1;01.13 onward. Initial and medial [ɫ], appearing mostly in a highly variable proto-word [ɫæ], [ʌɫʌ], [lɔʔ 'luə,] etc., modeled on “hello,” starts at 1;03.22. In the Later period, [k] appears (appropriately aspirated in initial position) at 1;06.18; [n], after having been marginally present since 1;01.13, takes a sudden jump at 1;07.10; and [p] appears, generally in word-final position, at 1;07.17. The observed order [d, l, k, n, p] elaborates Menn's original (1971) report that Jacob developed oral stop consonants in the order [d], then [k/g], and finally [p/b]; the development of labials after velars is somewhat unusual (found in only one English-acquiring child out of 66 by Stoel-Gammon, 1985).

### Changes in Accuracy of Segment Production Over Time: The Single Word “Down”

Sixty utterances of “down” transcribed in the MPMC are established as tokens of the word by audible context or written field notes. The variety of forms appears bewildering (see Table 1): fully accurate as well as very approximate productions are present in both the Early sessions (32 tokens) and the Later ones (28 tokens).

**Table 1:** Representative forms and uses of “down” over 6 months.

**Early period**				**Later period**			
**Session**	**Form, IPA**	**Retrieval**	**Context and usage notes**	**Session**	**Form, IPA**	**Retrieval**	**Context and usage notes**
1;01.13	dõ	LTM	Preceded by sharp inbreath, followed by sound of blocks falling	1;06.18	d$\overset{\sim }{æ}\overset{˜}{℧}$:	LTM	Sound of small object falling.
	dæwn	LTM			d∧m	LTM	
1;02.07	n$\overset{\sim }{æ}\overset{˜}{℧}$	IMIT-3	The first in a sequence; accompanies knocking block tower down.		d$\tilde{\varepsilon }$õ	LTM	
	d$\tilde{æ}\tilde{\wedge }$	IMIT	Accompanies knocking block tower down.		d∧m	SREP	
	'n$\tilde{\wedge }$w$\tilde{\wedge }$	IMIT	Accompanies knocking block tower down.		daw	LTM	Emphatic.
	d$\overset{\sim }{æ}\overset{˜}{℧}$	IMIT	Accompanies knocking block tower down.		do:	LTM	Emphatic, accompanied by thump of object thrown onto floor.
	d$\tilde{\wedge }$n	SREP	Accompanies knocking block tower down.		'dowo	SREP	Emphatic, accompanied by clatter of object thrown onto floor.
	d$\tilde{æ}$$\tilde{\text{w}}$	IMIT	Accompanies knocking block tower down.		'dæw$\underset{\circ }{\text{w}}$	SREP	Emphatic.
	Ɉjõ:	IMIT	Accompanies knocking block tower down.		dæw	SREP	Emphatic.
	dæwn	LTM	Accompanies knocking block tower down.		dæw	SREP	
1;02.26	ŋgɛ	LTM	Immediately precedes sound of infant-level Lego blocks falling.		do	LTM	
	dæ	IMIT	Sound of Lego blocks falling. Knocked over by either INV or CHI.		'etsi 'do	IMIT SREP	Imitating 'that's down'
	ɛd d_ɔ_	BB IMIT			d∧m:	SREP	
	dũ	LTM		1;07.10	dæ$\tilde{\text{w}}$n	LTM	Shouted.
	dũ:m	SREP		1;07.17	dæw	LTM	Question intonation; INV response “well, you can get down.”
	dæõ dɛp	LTM BB	NOT block game - CHI playing with pen cap. Intent unclear.		dæow	LTM	Question intonation; INV response “could put a block in the truck.”
	dum:	IMIT-1	INV: first you said down and then you knocked it down!		d$\tilde{\wedge }$m	LTM	Series of “down” utterances prefaced by note “CHI likes to knock block towers over but he wants INV to build them up.”
	dum	LTM			dæγm	SREP	These three utterances are apparently all CHI saying he wants a tower built so he can knock it over; no sound of falling blocks.
	d∧m	LTM			d$\tilde{æ}$$\tilde{\text{w}}$mn	SREP	
	$\underset{\circ }{\text{mmm}}$:	LTM	INV response: “yeah, it went down.” “Hat” fell off a toy egg after INV put it on.		d$\tilde{æ}$$\tilde{\text{w}}$n	SREP	
	dɛwn	IMIT			ɛn dæ$\tilde{\text{w}}$n	IMIT-5	New game of rolling a toy bus or truck up and down Jacob's leg, foot, etc.
	don	LTM	Intonation and pitch suggest narrative (“it fell down”).		dæw	LTM	Rises to squeal at peak around 1660 Hz.
	du:n	LTM	Intonation and pitch suggest narrative (“it fell down”).		d$\tilde{æ}$:	IMIT	Question intonation; seems to be verifying his understanding of INV's advice “easier to get (th)em to roll down.”
	'dı?auwn	IMIT			do	IMIT	
	dɩn	LTM	INV: “down, yeah, you down” (physical play)		d$\begin{array}{ll} \text{d}\tilde{æ} & \text{wwei}\\ & \circ \end{array}$	IMIT BB	Rolling toy truck down inclined top of large cardboard box.
1;03.22	dom	LTM	Not block game. CHI addressing CAM, who answers “yeah, down.”		Ɉjeã$\tilde{\text{w}}$ klaim	LTM LTM	Wants to climb onto the box. INV: “no, don't get on it, Jacob.”
	d℧m	IMIT	INV had said “it's upside-down,” referring to toy telephone.		dæ$\tilde{\text{o}}$ 'vi	IMIT BB	INV, slightly earlier: “ball went down.”
	næo	LTM	INV: yeah, it went down and then came out again (hammering-ball-into-box toy)		d$\tilde{æ}$$\tilde{\text{w}}$n	SREP-1	
	dn	LTM	INV: yeah, it went down (same toy)				
	da:m	LTM					
	d$\tilde{\wedge }$n	LTM	Possibly a response to “where's the ball?”				
	d℧m	LTM	After INV: “find the cookie in the bucket?”				
	Ɉæwn	LTM	Very emphatic, sounds like he threw a hard object onto the floor while saying it.				

*Key: LTM, retrieved from long term memory, no recent model form; IMIT, imitation of immediately preceding adult utterance; IMIT-n, imitation of adult utterance n adult turns back; SREP, self-repetition. BB, babble syllable accompanying “down.” INV, Investigator; CHI, Child; CAM, videocamera operator*.

The imitations (IMIT) appear at best slightly more accurate overall than the “spontaneous” (LTM) attempts (i.e., the tokens that Jacob must have retrieved from his long term memory because no one had recently uttered them). The initial [d] becomes more stable over time (accuracy 81% Early, 96% Later). The diphthong also improves (accuracy ignoring details encoded by diacritic 33% Early, 55% Later; accuracy counting diacritics, 11% Early, 21% Later). However, the final [n] deteriorates over the 6 months (31% correct Early, 18% Later).

### Articulatory Gestures as Precursors to the Emergence of Segments in Word Production: More About “Down”

Jacob's fine-grained variations in the forms for “down” resist analysis by ordered rules or ranked constraints. Such “unruliness” has long been noticed for the early months of speech (e.g., Ferguson and Farwell, 1975; Fikkert and Levelt, 2008); many children (but not all!—e.g., Ferguson et al., 1973; Menn and Vihman, 2011) begin speech production by attempting each word as a (more or less undigested) whole, rather than as a sequence of phonetic segments with appropriate coarticulations.

A segment like [t^h^] or [a] is typically defined as a bundle of articulatory features that co-occur simultaneously or in close sequence. But perceptually, a segment is also a bundle of co-occurring auditory/acoustic features. Children start to learn auditory representations before birth (Mehler et al., 1988), eventually inducing auditory-perceptual representations of segments from hearing thousands of examples of speech sounds: they register which features tend to co-occur and in what positions with respect to word and syllable boundaries (Pierrehumbert, 2003). An analogy may be helpful: finding the segments in the flow of input speech sounds is like finding the harmonic relations (chords) and chord progressions that are implicit in the flow of parallel voices in musical counterpoint.

With increasing motor maturation children start to learn articulatory representations, trying to reproduce some of the sounds they hear and often selecting adult models which match their own babble or earliest speech attempts (Vihman, 1993, 1996). They learn motorically which articulatory configurations reliably co-occur simultaneously or in close sequence (Beckman and Edwards, 2000; Edwards et al., 2004; Hay et al., 2004), and which articulatory features of sounds co-occur. Gradually, phonetic segments emerge (alongside whole words and syllables) as units of speech production—if not during late babble, then as vocabulary grows during the 1st months of speech (Walley, 1993; Menn and Vihman, 2011). Auditory and motor representations of a sound must eventually become tightly linked as part of development toward adult-like phonological representation.

Returning to Jacob's development, analyzing his attempts to say “down” in terms of articulator movements and vocal tract airflow instead of as attempts to string segments together gives us a mechanism for explaining the peculiar and frequent [m] at the end of his attempts at “down.” Analysis in terms of articulatory movements also enables us to be more precise about what it means to attack a word “as a whole,” and what it means for articulatory representations of segments to “emerge” from experience.

Without engaging in the technical apparatus of Gestural Phonology (Browman and Goldstein, 1986), consider an “articulatory gesture” intuitively, as a motion or continuous trajectory of a single articulator, for example “open the lips;” “raise the back of the tongue,” “flip the tongue tip up to the center of the hard palate and let it fall again,” “bring the vocal folds together loosely.” Jacob's various productions of “down” (Table 1) show that he is able to produce all the articulatory gestures needed to say the word. But like any beginner learning a complex motor sequence, he makes timing errors, and occasionally skips a gesture entirely.

The fact that Jacob uses *only* the relevant articulatory gestures shows that he knows what “down” sounds like; that is, he has a detailed auditory representation of the word as a whole.

**Phonetic details**. (Phoneticians and speech-language pathologists can skim this section; we have written it for colleagues and students who normally work at the lexical and syntactic levels and rarely need to deal with IPA or articulation. Readers with no background in phonetics should also see the Appendix (Supplementary Material), “How to say down”).

**Timing of velar lowering vs. tongue-blade raising**. In saying the word “down,” the velum must be lowered during the production of the diphthong [æw]. Many of the minor variations in Jacob's output come from small differences in his timing of this articulatory gesture; the sooner he lowers his velum, the more of the diphthong [æw] is nasalized, i.e., the greater the part of [æw] that is made with air flowing out both his nose and his mouth, resulting in [æ$\tilde{\text{w}}$] or [$\tilde{æ}\tilde{\text{w}}$]. All of these versions of the diphthong are acceptable in English.

**Timing errors in producing “down”**

Errors in velum movement:

1) If Jacob lowers his velum before or at the same time as he raises the blade of his tongue to make the initial [d], he produces initial [n], i.e., “noun” instead of “down.”
2) If he lowers his velum during the vowel without actually making the second tongue-to-gum-ridge contact needed for the final /n/, the result is an acceptable nasalized vowel, but without the nasal consonant that should follow it—i.e., the non-English form [d$\tilde{æ}$w].
3) When he does not get around to lowering his velum at all, he produces non-nasal forms like [dæw] (similar to the name “Dow”).

Other timing errors:

4) If Jacob starts rounding his lips and raising the back of his tongue for the [u] too soon, he produces forms like [dũn] (similar to “doon”). If in addition he misses the second tongue-to-gum-ridge contact for the final /n/, the output may be [do] (“dough”), or non-English [dõ], depending on whether he remembers to lower his velum.
5) If he forgets to keep his lips apart while raising his lower jaw for the final consonant, he produces forms like [dũm] (“doom”).
6) If he lingers too long on any of the articulatory configurations, he produces the forms that are heard as long vowels or consonants ([æ:], [o:], [m:]).

**Summary: what Jacob knows before motor knowledge of the segment [n] emerges**

Jacob has a good auditory representation of “down” —i.e., he knows in detail what it sounds like. However, his motor representation of it is missing some essential information: although the several articulatory gestures that his tongue and velum need to make for the segments in the word are established, their relative timing is poorly controlled.

The lack of coordination between the gestures of lowering the velum, raising the jaw, and raising the tongue blade implies, in particular, that the phonetic segment [n] is not yet his articulatory target for the end of the word “down.” Rather, he knows three separate pieces of information: that the word ends with a lowered velum (to produce nasalization) and that the word ends with raising the jaw and also raising the tongue blade (to make an alveolar closure). These three motor gestures and their relative timing have yet to be welded into a single unit that would constitute a motor representation for producing the alveolar nasal consonantal speech segment [n], let alone the auditory-motor complex that would constitute an adult-like representation of the speech sound [n].

Jacob's pre-segmental motor representation of word-final /n/ contrasts with his near-complete representation of both the auditory and motor aspects of word-initial /d/, as evidenced by the stability of his productions of the /d/ sound. Thus, he appears to be in transition from a pre-segmental to a segmental organization of his speech production for this word: although he appears to have a well-defined word-initial segment [d],^3^ the word-final segment [n] has not yet emerged as a unit of speech production^4^.

## The Potential of the MENN Phonetic Mini-Corpus

The MPMC contains multiple tokens of several other words that have not yet been analyzed for what they can tell us about the way phonological representations develop. These additional words should be helpful in evaluating the ways in which contemporary approaches to early phonological development such as the A-Map (Inkelas and Rose, 2007; McAllister Byun et al., 2016), the Linked-Attractor Model (Menn et al., 2013), and Template Theory (Vihman, 2019) complement one another. For children with “unruly” speech like Jacob, detailed articulatory analyses of multiple tokens of the same word over time enable us to create a richer picture of the mechanisms involved in development from early holistic auditory and articulatory representation toward a segmental as well as autosegmental phonological representation of the words in a speaker's lexicon.

## Data Availability Statement

The datasets generated for this study can be found in online repositories. The names of the repository/repositories and accession number (s) can be found at: https://phonbank.talkbank.org/derived/.

## Ethics Statement

Data were collected in 1974-75 with NSF funding. Ethics committee information is not available. Written informed consent to participate in this study was provided by the participants' legal guardian/next of kin.

## Author Contributions

LM is principally responsible for creating the corpus content and drafting the article. AP is responsible for choosing the sections to be transcribed and editing. YR is responsible for the Phon version of the data and the numerical analyses. All authors contributed to practical and theoretical discussions and to the final form of the document.

## Conflict of Interest

The authors declare that the research was conducted in the absence of any commercial or financial relationships that could be construed as a potential conflict of interest.

## Footnotes

^1^Praat (2020). Available online at: http://www.fon.hum.uva.nl/praat/ (accessed September 21, 2020).

^2^CLAN. Available online at: https://dali.talkbank.org/clan/.

^3^Jacob's control of the position where the front of his tongue must hit the roof of his mouth to form the initial [d] is not perfect: sometimes it hits a little too far back, resulting in the voiced palatal stop consonant [ɫ], a speech sound not used contrastively in English.

^4^Compare K's multiple attempts at “pen,” Ferguson and Farwell (1975, p. 423, fn. 8).
