# Supplementary material for: The Menn Phonetic Mini-Corpus: Articulatory Gestures as Precursors to the Emergence of Segments
Source: Front Psychol. 2021 Apr 28;12:646090. doi: 10.3389/fpsyg.2021.646090 (PMC8113676; doi:10.3389/fpsyg.2021.646090)
Supplement: Supplementary file 1 [file Data_Sheet_1.docx]

**Appendix**: **How to say ‘down’** (for readers with limited background in phonetics)

Compare Fig 1a and 1b: For [d] (Fig 1a), the velum (cartilaginous ‘soft’ palate) is in the raised position, closing off the nasal passage so air can’t flow out of the nos~~e~~. To say [n], in contrast, the velum is lowered like a trap door to an attic (Fig 1b), so air flows out of the nose but not the mouth.

Insert Fig. 1 – standard articulatory diagram for /d/ and /n/, 1a velum raised; 1b velum lowered.


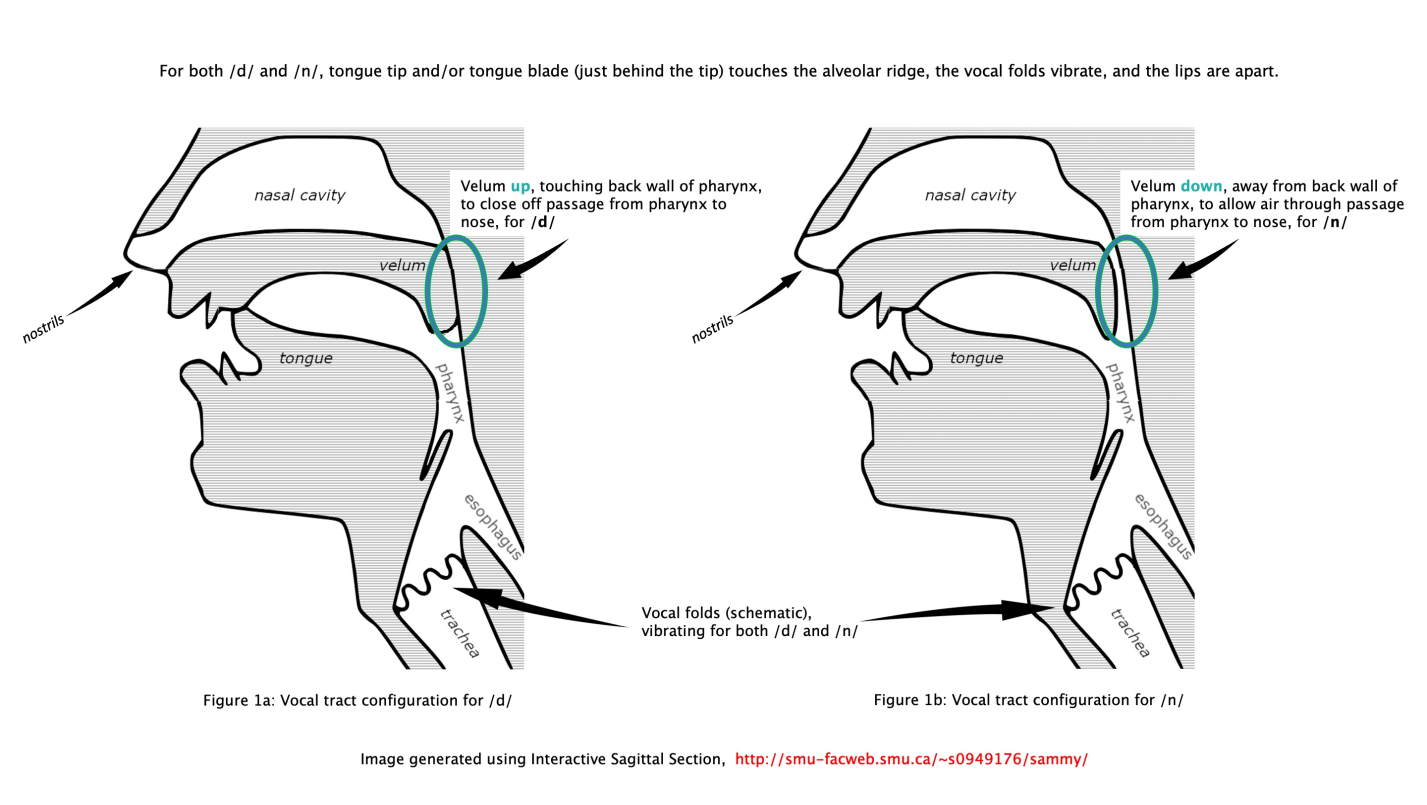


Sequence of gestures for saying ‘down’:

1. Start airflow from the lungs and raise the velum to block the nasal passage. To make a [d], raise the jaw and the tongue blade and edges so that the oral airflow is blocked at the alveolar ridge for a short time, say 40 to 60 msec. (When raising the jaw, don’t let the lower and upper lips touch; if you do, you’ll accidentally make a [b].) See Figure 1a.
2. Keep the velum raised and keep air flowing from the lungs into the mouth. During the period of alveolar airflow blockage or within 30msec. afterward, bring the vocal folds close together, so that airflow from the lungs into the mouth can make the vocal folds vibrate, completing the production of [d]. (If the vocal cord vibration is delayed longer than about 40msec, a [t] will result instead of a [d].) See Figure 1a.
3. Keep the vocal fold vibration going; lower the jaw, the lower lip, and the front part of the tongue so that the mouth is fairly far open, making the first part of the diphthong [æw], the vowel [æ] or [a] (the vowel of ‘cat’ or the vowel of Boston ‘father’). You may lower the velum now or at any time before step (5). If you do it at the beginning of the [æ], it creates a fully nasalized diphthong [æ̃w̃].
4. Keep the vocal fold vibration going. Pull the body of the tongue back and up towards the position needed for making [u], keeping the blade of the tongue low; at the same time, raise the jaw and purse the lips to bring them into the position for [u] or [w]. If you lower the velum during these actions, you will nasalize just the second part of the diphthong [æw̃].
5. After the jaw-lip-tongue motion of step 4) has gotten at least to an [o] shape if not all the way to [u], raise the tongue blade so that it contacts the alveolar ridge and blocks the air from flowing out of the mouth. Keep the velum down so the air continues to flow out of the nose and continues to make the vocal folds vibrate. Keep the lips apart. This will produce the [n]. See Figure 1b. (If you raise the tongue blade too slowly, the lip-pursing and jaw-raising will make the lips touch each other before the tongue blade hits the alveolar ridge, resulting in an [m] instead of an [n].)
